# Controllable Synthesis, Photocatalytic Property, and Mechanism of a Novel POM-Based Direct Z-Scheme Nano-Heterojunction α-Fe_2_O_3_/P_2_Mo_18_

**DOI:** 10.3390/molecules28186671

**Published:** 2023-09-18

**Authors:** Yanlin Zhang, Mingyu Zhao, Jubo Huang, Nan Zhao, Haihui Yu

**Affiliations:** School of Chemical Engineering, Northeast Electric Power University, Jilin 132012, China; yanlinzhang70@163.com (Y.Z.); 16645796988@163.com (M.Z.); 18755787591@163.com (J.H.); zn991214@163.com (N.Z.)

**Keywords:** POM-based photocatalyst, nano-heterojunction, direct Z-scheme, controllable synthesis

## Abstract

In order to improve photocatalytic activity and maximize solar energy use, a new composite material Fe_2_O_3_/P_2_Mo_18_ was prepared by combining polyoxometalates (P_2_Mo_18_) with Fe_2_O_3_ nanosheets. FT-IR, XRD, XPS, SEM, TEM, UV-vis, EIS, and PL were used to characterize the composite material, and nano-Fe_2_O_3_ of different sizes and morphologies with a controllable absorption range was prepared by adjusting the reaction time, and, when combined with P_2_Mo_18_, a composite photocatalyst with efficient visible light response and photocatalytic activity was constructed. The EIS, Bode, and PL spectra analysis results show that the Fe_2_O_3/_P_2_Mo_18_ composite material has outstanding interfacial charge transfer efficiency and potential photocatalytic application possibilities. Model reactions of methylene blue (MB) and Cr (VI) photodegradation were used to evaluate the redox activity of Fe_2_O_3_/P_2_Mo_18_ composites under simulated visible light. The photocatalytic degradation rate was as high as 98.98% for MB and 96.86% for Cr (VI) when the composite ratio was Fe_2_O_3_/P_2_Mo_18_-5%. This research opens up a new avenue for the development of high-performance photocatalysts.

## 1. Introduction

Environmental changes are becoming increasingly significant as business develops, and environmental protection has been at the center of scientists’ attention. Researchers are committed to exploring the usage of clean energy in the face of environmental concerns. Photocatalysis technology has been widely used in many fields in recent years, including in photocatalytic hydrogen production [1], photocatalytic carbon dioxide production of organic matter [2], photocatalytic degradation of nitrate [3], photocatalytic synthesis of ammonia [4], and pollutant degradation [5]. One of the most efficient approaches to tackling environmental problems is to employ photocatalysts to degrade organic contaminants in water using sunlight [6,7,8]. However, photocatalysis research focuses on how to make photocatalysts more stable and efficient.

Single-component photocatalysts have been observed to have low optical absorption, poor photostability, and significant charge recombination. Using two photocatalysts to construct a heterojunction can give a new path for charge transfer, successfully solving the abovementioned concerns. Because of their unique charge transfer paths, Z-scheme heterojunctions are commonly thought to offer tremendous promise for enhancing photocatalytic performance compared to conventional type-I and type-II heterojunctions. Not only is charge transfer efficiency quicker in Z-scheme heterojunctions, but they also do not require costly redox mediators. They may retain the most significant redox potential of the components. Based on the unique Z-scheme heterojunction structure, the catalytic performance improves remarkably. For example, the ZnIn_2_S_4_/ZrO_2_ composite photocatalyst generated using the photothermal method may considerably increase photocatalytic hydrogen generation performance [9]. Electrostatic assembly was used to create the CeO_2_/WO_3_ Z-scheme heterojunction, which has a higher CO_2_ reduction capacity in visible light due to its good charge separation efficiency [10].

POMs, or polyoxometalates, are metal oxide nanoclusters with specific structures composed of heteroatoms and ligand atoms [11]. They have distinct physical and chemical characteristics due to their structure, such as acid–base and redox properties, making them ideal materials for photocatalysts [12], medical care [13], and magnetic materials [14]. Generally, POMs absorb UV and near-UV light strongly and undertake efficient intramolecular charge transfer in response to light irradiation [15]. In particular, during photo-redox reactions, POM frameworks are usually stable and may reversibly transfer electrons across various substrates while maintaining their structure [16]. Meanwhile, the redox potential of POMs may be modified at the molecular level by changing their structure and constituent elements or by adding heterometal atoms or cations into their skeleton, altering their redox capacity [17]. The POMs may accept several electrons into their structure, thus making multi-electron redox reactions possible, which are essential for the photocatalytic reactions that transform light energy into chemical energy [18].

Iron-based nanomaterials are essential in nanotechnology and have sparked great research interest. Nano-sized Fe_2_O_3_, which is highly cost-effective and has excellent performance among other compelling characteristics, is a particular standout. Fe_2_O_3_ is perhaps the most extensively utilized substance in iron oxides due to its variable shape. As we know, each of the four well-known crystalline Fe_2_O_3_ polymorphs (α-, β-, γ-, and ε-Fe_2_O_3_) has distinct characteristics and is helpful in a variety of fields [19,20,21,22]. Because of its low cost, facile preparation, environmental friendliness, and excellent chemical stability as a narrow-band (1.9–2.2 eV) n-type semiconductor [23], Fe_2_O_3_ has been intensively studied in various fields, including for use in catalysts [24], pigments [25], water treatmen t [26], magnetic materials [27], sensors [28], and lithium-ion batteries [29]. Typically, γ-Fe_2_O_3_ has been introduced in the field of photocatalysts, giving rise to composite materials with magnetic features and enhancing the separation of photo-induced carriers [30]. In recent years, a series of α-Fe_2_O_3_ nanoparticles with different morphologies has been synthesized using sol-gel [31], low-temperature calcination [32], and hydrothermal methods [33]. Although Fe_2_O_3_ has a broad absorption range, it is unsuitable for use as an ideal photocatalyst due to the difficulties in producing electron-hole pairs. Combining α-Fe_2_O_3_ with another semiconductor with adequate valence and conduction band positions is possible. Therefore, the preparation of synthetic composite materials using Fe_2_O_3_ and other materials is being extensively researched. The hydrothermal 3D SnO_2_/α-Fe_2_O_3_ nanocomposites and ceramics synthesized using Fe_2_O_3_/TiO_2_ composites exhibit excellent visible-light degradation capacities [34].

At the present stage, POMs with various structural types are now combined with a range of metal nanoparticles to generate a variety of heterojunction materials. According to band theory, the band gap of POMs is more than 2.4 eV, whereas that of transition metal oxides is typically between 1.7 and 3.2 eV [35,36,37]. Thus, combining POMs with transition metal oxide nanomaterials can significantly reduce the recombination likelihood of photogenerated electrons and holes, thereby improving photocatalytic performance. So far, much study has focused on Keggin-type POMs, while Dawson-type POMs have received less attention in photocatalysis. Dawson-type POMs outperform Keggin-type POMs regarding absorption range and photocatalytic activity sites. In particular, reports on Fe_2_O_3_ and Dawson-type POM combinations are exceedingly rare [38,39]. This study prepares a new direct Z-scheme photocatalyst Fe_2_O_3_/P_2_Mo_18_ using a “step-by-step” strategy by loading an as-prepared Fe_2_O_3_ nanosheet onto a P_2_Mo_18_ POM. According to the characterization and experimental results, the Fe_2_O_3_/P_2_Mo_18_-5% composites exhibit remarkable photocatalytic performance and photostability in both photocatalytic organic pollutant methylene blue (MB), an organic pollutant, and heavy metal Cr(VI), respectively. The probable charge transfer mechanism was then studied based on the active species trapping experimental results.

## 2. Results and Discussion

### 2.1. Structural Analysis and Physical Properties

**Figure 1:**
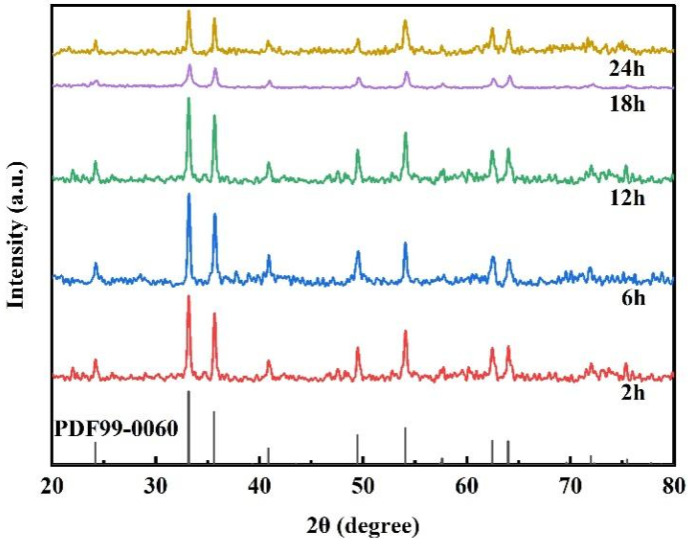
The XRD patterns of Fe_2_O_3_ nanoparticles.

The crystal structure of the Fe_2_O_3_ nanoparticles was characterized using X-ray diffraction (XRD), as shown in Figure 1. The diffraction patterns located at 2θ of 24.2°, 33.2°, 35.8°, 40.9°, 49.4°, 54.2°, 62.7°, and 64.0° are typical of the Fe_2_O_3_ (PDF#99-0060) characteristic diffraction peaks, corresponding to the (0 1 2), (0 2 4), (1 0 4), (1 1 0), (1 1 3), (1 1 6), (2 1 4), and (3 0 0) crystal planes, respectively [40]. Furthermore, no diffraction peaks of other compounds were identified in Figure 1, demonstrating that the purity phase of Fe_2_O_3_ was well prepared. During the synthesis, it was discovered that the reaction time significantly impacted the crystallinity. When the reaction time varied from 2 h to 12 h, the diffraction peak intensity of Fe_2_O_3_ steadily increased as the reaction time increased, as did the crystallinity. When the reaction time was between 18 h and 24 h, the diffraction peak intensity of Fe_2_O_3_ declined as the reaction time increased, as did the crystallinity; however, when the reaction time was 12 h, the prepared Fe_2_O_3_ exhibited the most incredible diffraction peak intensity and crystallinity.

**Figure 2:**
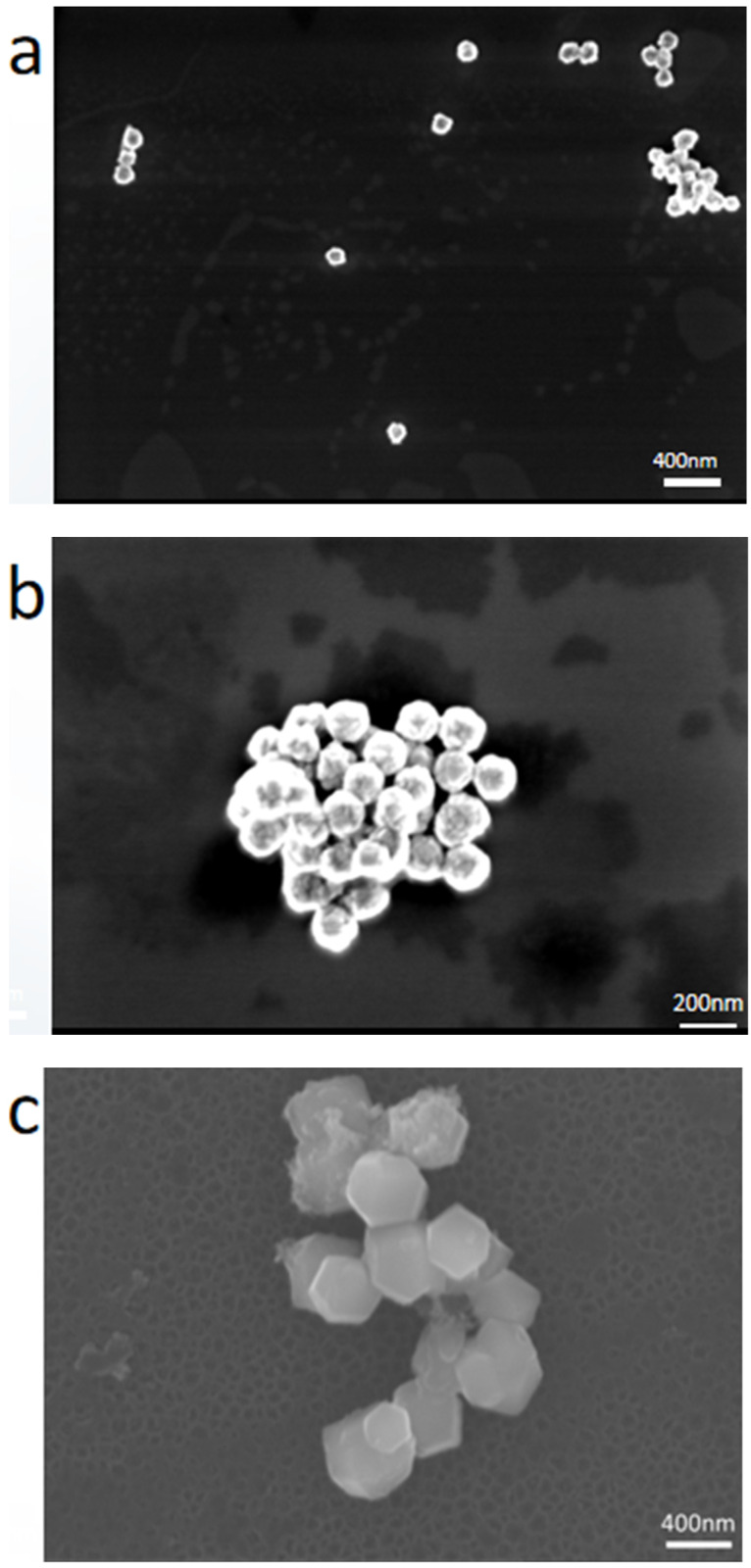

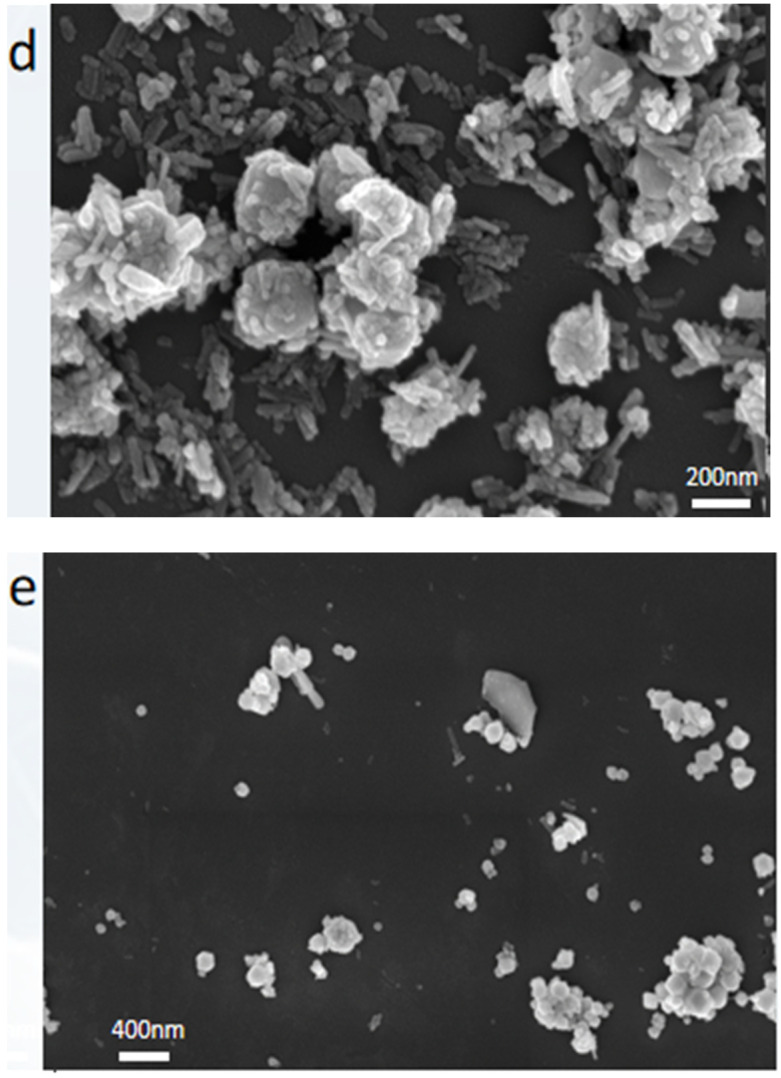
SEM images of Fe_2_O_3_ at different reaction times ((**a**) 2 h, (**b**) 6 h, (**c**) 12 h, (**d**) 18 h, (**e**) 24 h).

Then, SEM was used to measure and study further the influence of synthesis time on the physicochemical parameters of Fe_2_O_3_, as shown in Figure 2. The Fe_2_O_3_ formed after a 2-h reaction period was homogenous spheres with a diameter of about 50 nm (Figure 2a). The diameter of the Fe_2_O_3_ nanoparticles rapidly increased to about 100 nm after 6 h of reaction time (Figure 2b). After extending the reaction period to 12 h (Figure 2c), hexagonal sheets of Fe_2_O_3_ nanoparticles with unique morphologies were produced. The reaction period was gradually increased to 18 h and 24 h (Figure 2d,e), and a mixed state of rod and bulk Fe_2_O_3_ was formed. The UV-vis spectra of the Fe_2_O_3_ with varying reaction periods were used to investigate the influence of reaction time on the light absorption capabilities (seen in Figure S1); the analysis results show that the UV absorption ranges of the five samples were all in the range of 200–600 nm, and the reaction period of 12 h produced the maximum light absorption intensity and the widest visible-light absorption range of Fe_2_O_3_. The preceding results demonstrated that the ideal reaction period of the Fe_2_O_3_ produced was 12 h.

Based on the preceding findings, the FI-IR spectra of the Fe_2_O_3_/P_2_Mo_18_-x (x = 1, 3, 5, 8, 10) composites with different ratios of Fe_2_O_3_ under a reaction time of 12 h are depicted in Figure S2. The characteristic absorption bands of P_2_Mo_18_ are located at 700–1100 cm^−1^, with the typical peaks at 1078 cm^−1^ and 1002 cm^−1^ attributed to P-O. The slight shift of the characteristic peaks due to vibrational splitting caused by the different spacing of P and O in PO_4_, and 939 cm^−1^, 905 cm^−1^, and 777 cm^−1^ are attributed to the Mo=Oa tensile vibration, the tensile vibration of the Mo-Ob-Mo bond, and the bending vibration of Mo-O_c_-Mo, respectively [41]. The peaks at 2955 cm^−1^, 2876 cm^−1^, and 1462 cm^−1^ are attributed to the asymmetric and symmetric telescoping vibrations of C-H, N-H, and C-N telescoping vibrations of tetrabutylammonium bromide, respectively [42]. When Fe_2_O_3_ was composited with P_2_Mo_18_, the distinctive peaks of P_2_Mo_18_ in the composite showed a weak shift compared to pure P_2_Mo_18_, indicating that the binary materials interacted.

**Figure 3:**
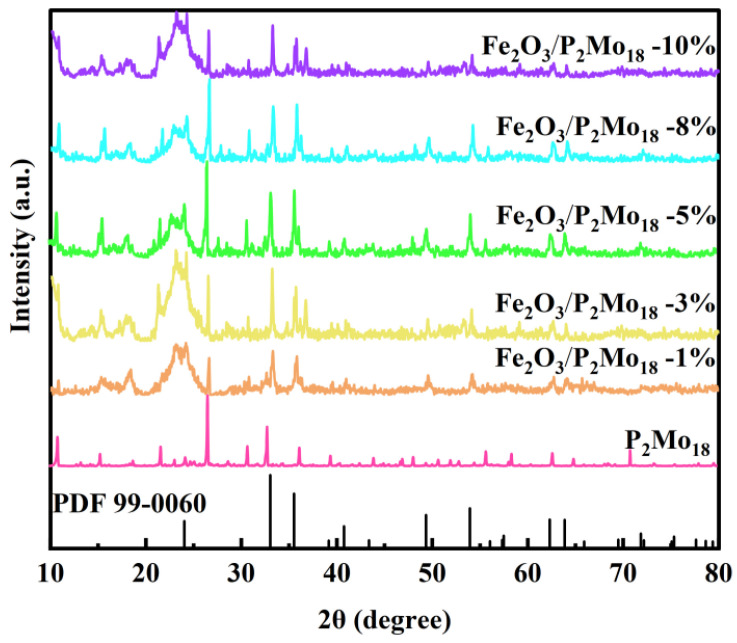
XRD pattern of composite Fe_2_O_3_/P_2_Mo_18_.

XRD characterization was performed to determine the crystalline structure and phase composition of the sample. As shown in Figure 3, the crystal structure of the produced Fe_2_O_3_/P_2_Mo_18_-x samples with different ratios of Fe_2_O_3_ was investigated. When the X-ray powder diffraction patterns of the composites are compared to the typical diffraction of Fe_2_O_3_ located at 24.2°, 33.2°, 35.8°, 40.9°, 49.4°, 54.2°, 62.7°, and 64.0°, the patterns of these samples show similar characteristics before and after assembly except for the additional peaks of the P_2_Mo_18_ polyoxoanion, confirming that the structures of the Fe_2_O_3_ nanosheet and polyoxoanion P_2_Mo_18_ are stable during the compositing process.

**Figure 4:**
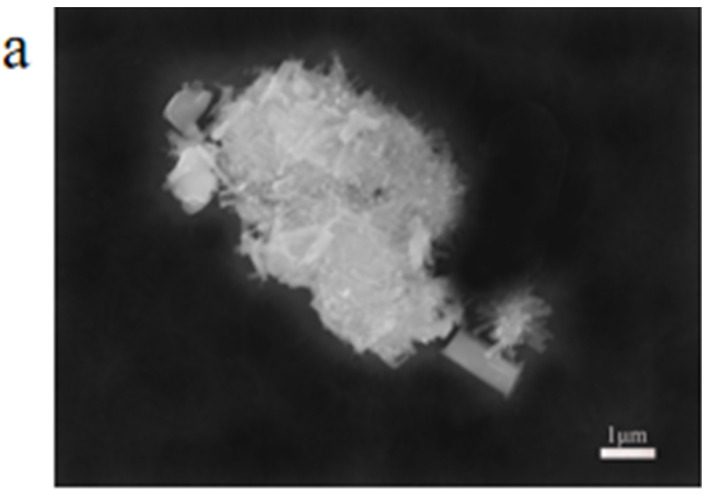

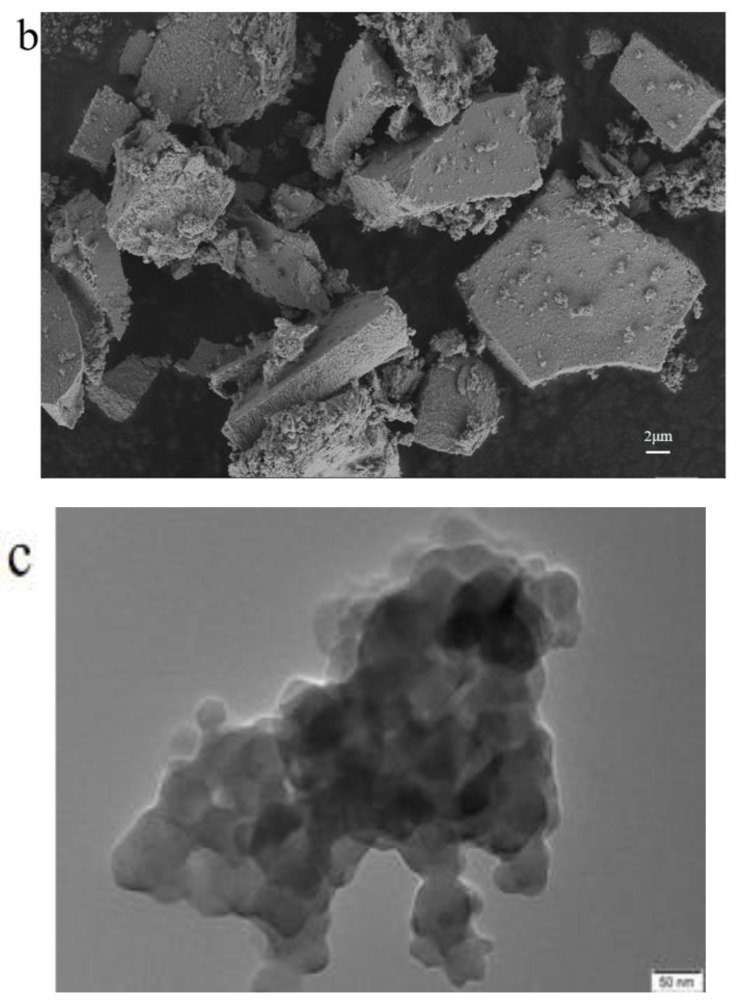
SEM image of P_2_Mo_18_ (**a**), SEM (**b**) and TEM (**c**) images of Fe_2_O_3_/P_2_Mo_18_.

Scanning electron microscopy (SEM) and transmission electron microscopy (TEM) were used to examine the morphology and microstructure of the composite catalysts, as shown in Figure 4. Figure 4a shows an SEM picture of P_2_Mo_18,_ where the morphology of P_2_Mo_18_ is mainly in the form of rods and blocks that agglomerate to produce a cluster-like condition. Figure 4b shows an SEM picture of a Fe_2_O_3_/P_2_Mo_18_ sample. The kinetic management of the crystal development process by the surfactant TBAB, which influences the crystals’ direction and growth rate, may be responsible for the modification of the POM shape in the composites. Figure S3 presents the isothermal adsorption–desorption curves and the corresponding pore—size distribution curves of Fe_2_O_3_/P_2_Mo_18_. The results show that the BET surface area of the composites is 49 m^2^/g and the average pore diameter is 21 nm. Then, the TEM analysis was used to examine the microstructure of the composites. As shown in Figure 4c, obvious lattice spacers and two-bit interfaces indicate that the composite material Fe_2_O_3_/P_2_Mo_18_ was effectively manufactured. On the P_2_Mo_18_ surface, the Fe_2_O_3_ forms synergistic interfacial contacts, and the resultant heterojunction may decrease the compounding rate of photogenerated carriers and increase the photocatalytic efficacy of the composite material.

**Figure 5:**
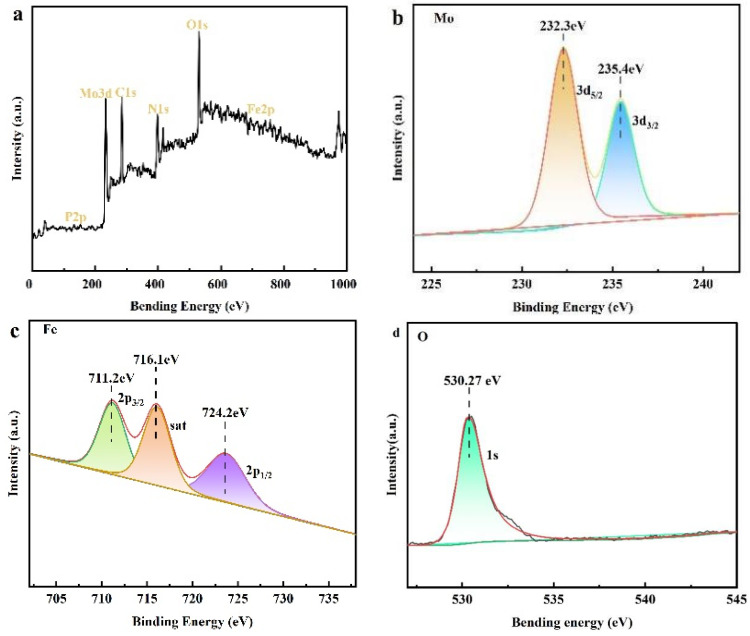
XPS spectra of Fe_2_O_3_/P_2_Mo_18_. Survey (**a**), Mo 3d (**b**), Fe 2p (**c**), and O1s (**d**).

To validate that the composites were successfully synthesized, X-ray photoelectron spectroscopy (XPS) was utilized to examine the chemical composition and elemental valence states of the obtained Fe_2_O_3_/P_2_Mo_18_ nanocomposites, as shown in Figure 5. The presence of the components P, Mo, C, N, O, and Fe throughout the XPS survey spectra of the composite, which is consistent with the composition, suggests that the synthesis is effective. Figure 5b depicts the nuclear energy level spectra of the Mo 3d spin-orbital. The distinctive peaks located at 232.3 eV and 235.4 eV correspond to Mo3d_5/2_ and 3d_3/2_ of Mo^6+^, respectively [43]. The nuclear energy level spectra of the Fe 2p spin-orbital are shown in Figure 5c. The spectra were separated into three binding energies: 711.02 eV, 716.1 eV, and 724.2 eV, and each distinctive peak is in good agreement with 2p_3/2_, sat, and 2p_1/2_ for Fe^3+^ [44], respectively. The O1s spectrum (Figure 5d) located at 530.27 eV is attributed to the lattice oxygen of the sample [45].

**Figure 6:**
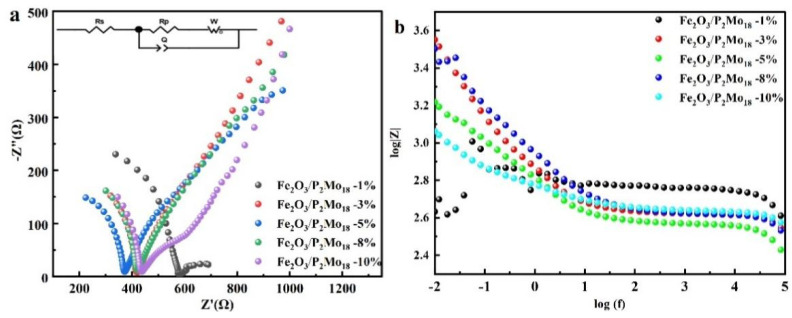
Nyquist (**a**) and Bode (**b**) plots for different ratios of Fe_2_O_3_/P_2_Mo_18_.

The electrochemical characteristics and PL spectra were used to study the composites’ carrier transport and separation efficiency. An appropriate composite ratio was established via electrochemical and PL characterizations of the composite Fe_2_O_3_/P_2_Mo_18_. As shown in Figure 6a, Fe_2_O_3_/P_2_Mo_18_-5% has a smaller arc radius in the high-frequency region than other composites, indicating that the material has a favorable charge-transfer resistance during the photogenerated electron transfer process. As a result, adding 5% Fe_2_O_3_ enhances the interfacial charge transfer and boosts the prospective photocatalytic activity. According to the Bode diagram in Figure 6b, the |Z| values of Fe_2_O_3_/P_2_Mo_18_-5% are lower than the other ratios, indicating that the electrons have a greater electron transfer rate. The development of a heterojunction creates an entirely new charge transfer route, facilitating the separation and transport of photogenerated electron-hole pairs.

**Figure 7:**
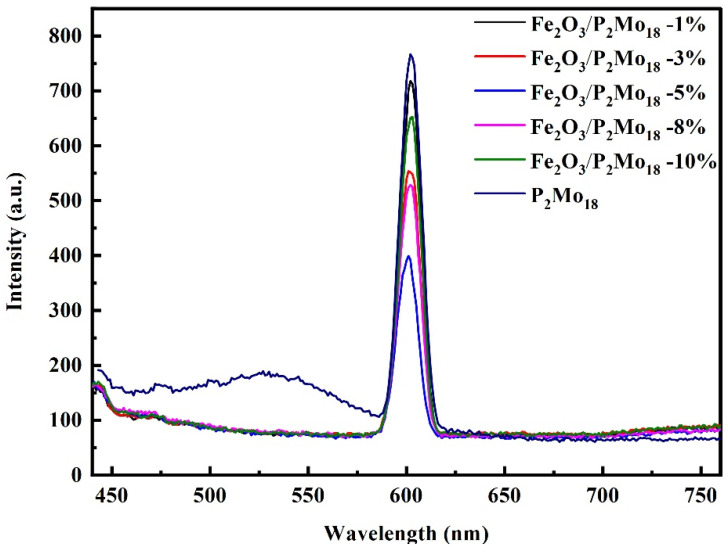
Photoluminescence spectra of Fe_2_O_3_/P_2_Mo_18_.

Meanwhile, PL spectroscopy confirmed the photoinduced carriers’ charge recombination and migration efficiency in the composite Fe_2_O_3_/P_2_Mo_18_. As we know, adding a modest proportion of Fe_2_O_3_ to the catalyst surface can create active sites. However, an excessive combined ratio can become a compounding center of photogenerated electrons and holes. Hence, several substances’ fluorescence emission intensity responses were tested for further characterization. A more significant photogenerated electron-hole pair complexation rate generally signifies a higher luminescence intensity of the activated catalyst [46]. The luminescence peaks at 600 nm for composites made using varied Fe_2_O_3_ mass ratios are compared in Figure 7. It is shown that the polyoxoanion P_2_Mo_18_ has the highest PL intensity due to its intrinsic photogenerated electron-hole pair complexation. Fe_2_O_3_/P_2_Mo_18_-5%, on the other hand, had a lower fluorescence peak intensity than those of different mass ratios, indicating that it was the most efficient in suppressing photogenerated carrier recombination. The EIS and PL analysis results show that the composite Fe_2_O_3_/P_2_Mo_18_-5% has the most effective photogenerated electron-hole pair separation and the fastest interfacial charge transfer rate.

**Figure 8:**
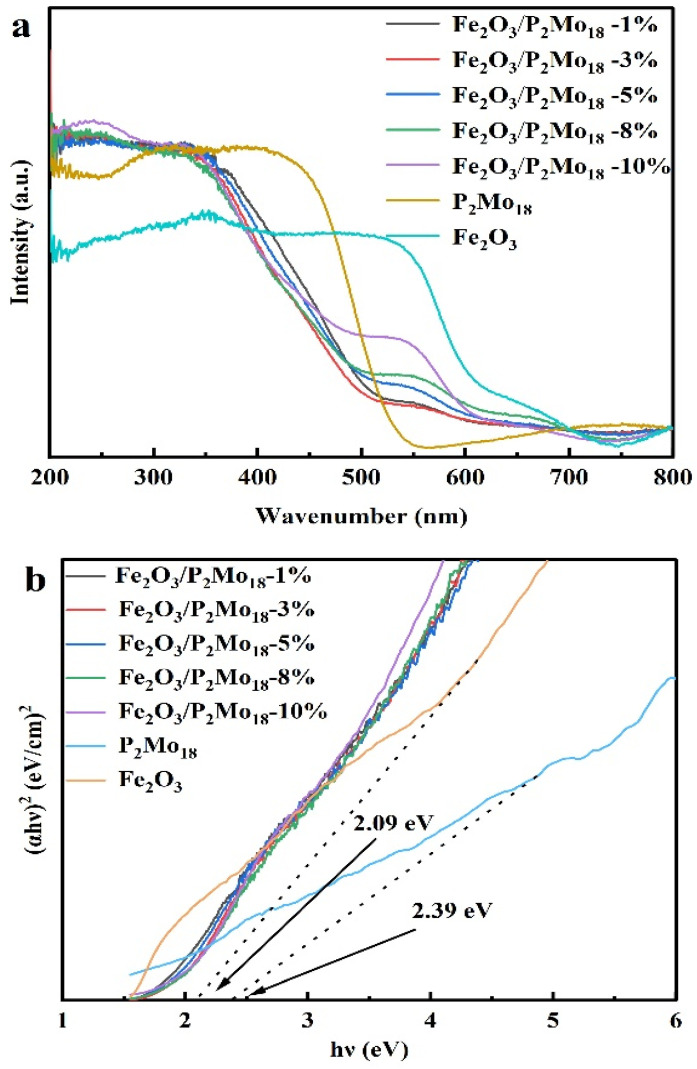
(**a**) UV−vis−NIR absorption spectra and (**b**) the corresponding Tauc plots [i.e., (αhν)^2^ vs. (hν)] of all of the as-prepared samples.

The UV-vis absorption spectra of the P_2_Mo_18_, Fe_2_O_3_, and Fe_2_O_3_/P_2_Mo_18_-x samples are given in Figure 8 to assess the optical absorption characteristics and optical band gaps of the produced photocatalysts. The primary absorption peaks of P_2_Mo_18_ emerged in the 200–550 nm range, which has a high solar-light use rate. While Fe_2_O_3_ has a light absorption range of 200–700 nm, Fe_2_O_3_ may extend the range to yellow-green or red light, considerably improving sunlight usage in the photocatalytic process. The absorption peak at 500–700 nm increases gradually with the increase in the Fe_2_O_3_ mass ratio in the figure for the composite material Fe_2_O_3_/P_2_Mo_18_-x, indicating that the composite material of Fe_2_O_3_ and P_2_Mo_18_ can effectively stretch the wavelength up to about 700 nm, which is conducive to the photocatalytic process. It suggests that the addition of Fe_2_O_3_ can significantly increase the composite catalyst’s visible-light usage. The band gap of the sample was calculated using the Tauc equation: (αhν)^1/n^ = A (hν − Eg), where α, h, ν, A, and Eg indicate the absorption coefficient, Planck’s constant, optical frequency, scaling factor, and energy band gap, respectively. Furthermore, the exponent is connected to the material’s inter-band hopping characteristics, which is 0.5 for direct semiconductors and 2 for indirect semiconductors. Based on the preceding equations, a plot of (αhν)^2^ versus (hν) is displayed in Figure 8b, and the bandgap values for the P_2_Mo_18_ and Fe_2_O_3_ samples are around 2.39 eV and 2.09 eV, respectively.

Additionally, to further investigate the influence of Fe_2_O_3_ with varied reaction times on the composites, Fe_2_O_3_ and P_2_Mo_18_ with varying reaction times were combined at an ideal composite ratio of 5%. The distinctive absorption band of the manufactured composite Fe_2_O_3_/ P_2_Mo_18_ occurs at 700–1100 cm^−1^ in the FT-IR spectrogram (Figure S4), which shows that the composite has little effect on the P_2_Mo_18_ characteristic absorption peak. In the UV-visible spectrum (Figure S5), because the peak intensity of Fe_2_O_3_ synthesized at 12 h was the greatest, P_2_Mo_18_ had the highest absorption peak intensity when compounded with Fe_2_O_3_ prepared at 12 h, and the composite performed best. The separation and transfer efficiency of photogenerated e^−^ and h^+^ may be explained using electrochemical AC impedance Nyquist plots and Bode graphs. The radius of the circle of the composite created using a reaction time of 2 h for Fe_2_O_3_ is more significant than that of the composite prepared using a reaction time of 6 h, as shown in Figure S6a, among the composites with the same ratio of Fe_2_O_3_. This is mainly because the particle size of the spherical Fe_2_O_3_ generated after 6 h is more significant than that of the spherical Fe_2_O_3_ prepared after 2 h. As a result, the smaller diameter of Fe_2_O_3_ formed at 2 h is more prone to aggregation, resulting in an electrostatic shielding effect and a higher resistance. In contrast, the Fe_2_O_3_ generated after 12 h has a consistently sized and structured sheet structure that is less prone to aggregation and has the lowest charge transfer resistance. The Fe_2_O_3_ morphology altered further as the reaction time increased, and the Fe_2_O_3_ morphology formed at 18 h was not uniform, with both rod-like and bulk-like morphologies that increased charge transfer resistance. When the reaction period approaches 24 h, a more fragmented bulk Fe_2_O_3_ is generated, increasing the charge transfer resistance. By modeling a circuit, the electrochemical impedance values are fitted, and the resistance of the circuit elements is employed to indicate the impedance magnitude of the composite material. According to the data, the resistance of the composites was 533.4 Ω at a reaction time of 2 h for Fe_2_O_3_, 494.6 Ω at a reaction time of 6 h, 274.9 Ω at a reaction time of 12 h, 428.9 Ω at a reaction time of 18 h, and 496.7 Ω at a reaction time of 24 h, which meant that the Fe_2_O_3_-12 h possessed the lowest internal resistance. The lower the modal value of the Bode plot, the better the material’s performance. According to the graph (Figure S6b), the composites composited with Fe_2_O_3_ and P_2_Mo_18_ at different reaction periods formed the composites with the lowest modulus values and the highest performance. Meanwhile, the PL spectrum reflecting the ability of photogenerated e^−^ and h^+^ complexation was recorded and is shown in Figure S7. The figure demonstrates that the composite formed by mixing Fe_2_O_3_ and P_2_Mo_18_ for 12 h is the most effective at suppressing photogenerated carrier complexes and has the best photocatalytic effect. According to the combined results, the composite Fe_2_O_3_/P_2_Mo_18_-12 h exhibits the most effective photogenerated electron-hole pair separation and the fastest interfacial charge transfer rate.

### 2.2. Photocatalytic Activity

**Figure 9:**
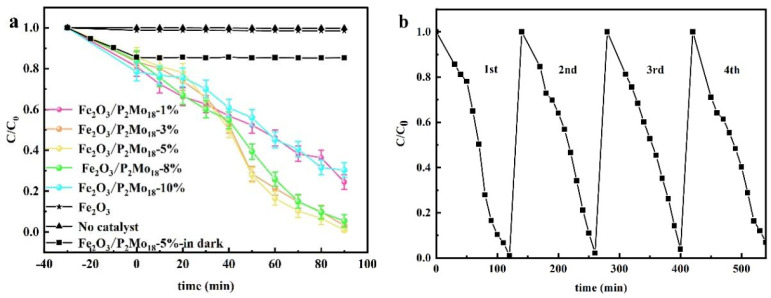

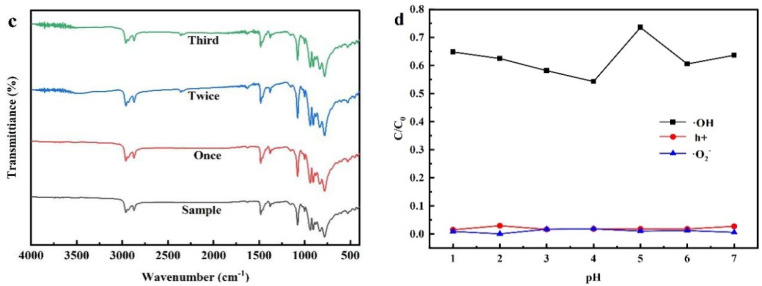
MB (20 mg/L) degradation of Fe_2_O_3_/P_2_Mo_18_ at different composite ratios (100 mg, 18.085 mW/cm^2^) (**a**), Fe_2_O_3_/P_2_Mo_18_ cycling experimental data (**b**), infrared comparison of composite cycling experimental samples (**c**), and photocatalytic mechanism test data (**d**).

MB was the target pollutant used to assess the composites’ photocatalytic efficacy. Figure 9a depicts the degradation efficiencies of various composite mass ratios. A series of composite samples generated with the addition of P_2_Mo_18_ were much more effective in degrading MB in visible light than catalyst-free and Fe_2_O_3_ alone, with the greatest photocatalytic effect of Fe_2_O_3_/P_2_Mo_18_-5% reaching about 98.98%. The photocatalytic cycling experiment is shown in Figure 9b, and it is seen that the degradation rate may reach 94% after the third catalytic experiment, and the degradation rate is somewhat lowered but stays constant with catalytic solid activity. In Figure 9c, we can see that the infrared characteristic peaks of the composites after and before activation are consistent, indicating that the photocatalyst is more stable.

As reported, multiple active compounds are frequently involved in the photocatalytic process of MB. Analyzing the critical active chemicals involved in the catalytic reaction, therefore, aids in investigating the photocatalytic reaction mechanism. At different pH levels, isopropanol, triethanolamine, and p-benzoquinone were used as scavenger agents for hydroxyl radicals (·OH), superoxide radicals (·O_2_^−^), and holes (h^+^), respectively, in the capture tests. The results are presented in Figure 9d. The graph’s general trend indicates that the addition of isopropanol has a considerable influence on the photocatalytic degradation of MB. After 90 min of light exposure, the degradation rate of MB by the composite Fe_2_O_3_/P_2_Mo_18_ was only about 40%. The results showed that the ·OH active ingredient was crucial in the photocatalytic MB breakdown. At pH < 4, the scavenger agent’s capacity to photocatalytically degrade MB progressively diminished with increasing pH, which was ascribed to a drop in hydrogen ion concentration, resulting in a weakening of the inhibitory effect of ·OH and a gradual rise in ·OH generation. The largest amount of ·OH was produced when the pH approached 5. However, a coupling reaction occurs between adjacent ·OH in solution, which consumes part of the active group ·OH [47]. The formation of ·OH diminishes slightly after pH > 6, but the coupling process between ·OH is decreased, enhancing the degrading impact.

**Figure 10:**
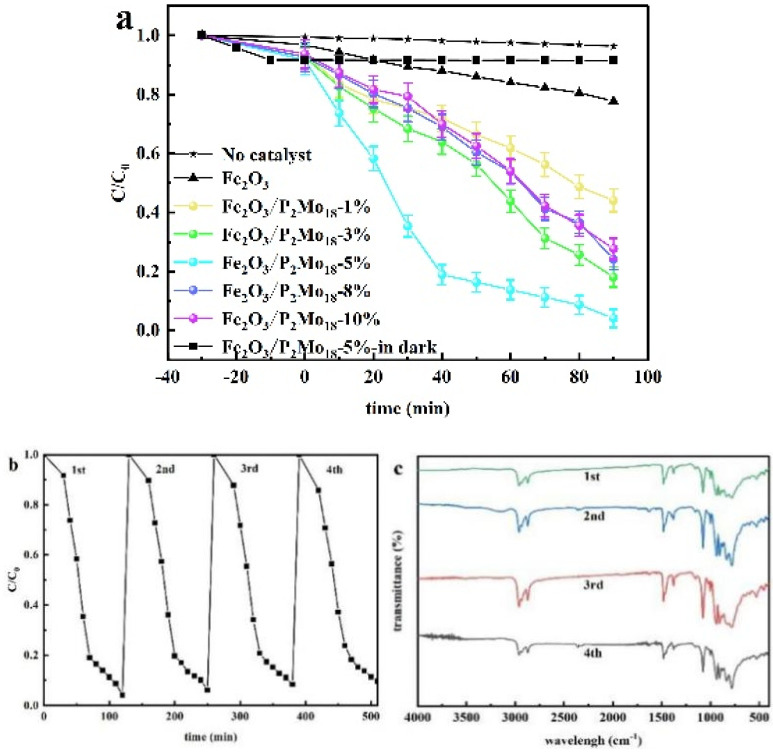
Degradation of Cr (VI) (50 mg/L) by Fe_2_O_3_/P_2_Mo_18_ at different composite ratios (100 mg, 18.085 mW/cm^2^) (**a**), photocatalytic cycling experimental data (**b**), and infrared spectra of catalyst cycling (**c**).

To replicate industrial Cr (VI)-containing wastewater and test the reduction ability of various composites to (Cr) under visible-light irradiation, an aqueous potassium dichromate solution at a concentration of 50 mg/L was utilized. Figure 10a depicts the concentration of Cr (VI) as a function of light-time, where C_0_ is the starting concentration of Cr (VI) and C represents the real-time concentration of Cr (VI). After 90 min of visible-light irradiation, the composite material Fe_2_O_3_/P_2_Mo_18_ had a significant degradation effect on Cr (VI) under light, and the photocatalytic effect of Fe_2_O_3_/P_2_Mo_18_-5% was better than that of others, reaching 95.86%. In addition, the photocatalytic test on the composite Fe_2_O_3_/P_2_Mo_18_-5% sample was performed for four cycles to evaluate the stability of the catalyst in the reduction of Cr (VI). As shown in Figure 10b, the degradation rate of Cr (VI) by the composite Fe_2_O_3_/P_2_Mo_18_-5% sample could still reach 90.43% after four cycles, despite a 5.43% drop. The consistency of the IR characteristic peaks (Figure 10c) between the composites after and before activation shows that the photocatalyst has better stability.

### 2.3. Mechanism

**Figure 11:**
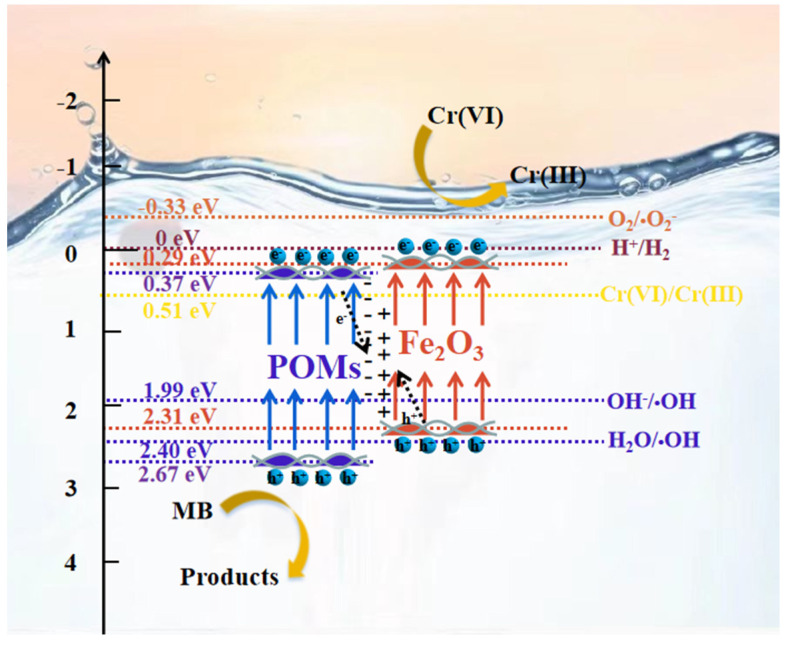
Schematic energy band structure of Fe_2_O_3_/P_2_Mo_18_ composites and photocatalytic reaction mechanism and charge transfer pathway for MB degradation and Cr (VI) reduction on Fe_2_O_3_/P_2_Mo_18_ heterojunction.

A proper energy band gap is required for the fabrication of heterojunctions. The cyclic voltammograms of P_2_Mo_18_ and Fe_2_O_3_ (Figure S8) show that the LUMO position of P_2_Mo_18_ is 0.37 eV and the CB position of Fe_2_O_3_ is 0.29 eV (concerning the normal glycogen electrode). The HOMO of P_2_Mo_18_ was calculated to be 2.67 eV, the VB position of Fe_2_O_3_ was estimated to be 2.31 eV, and the staggered energy band structure laid the groundwork for the formation of heterojunctions between the two components. The energy band structures of P_2_Mo_18_ and Fe_2_O_3,_ as well as the redox potentials of different reaction processes, are displayed in Figure 11 to examine the photocatalytic reaction mechanism and further define the heterojunction type and photogenerated electron transfer mode. When excited by visible light, photogenerated electrons of P_2_Mo_18_ on HOMO leap to LUMO, leaving photogenerated holes on HOMO. Because Fe_2_O_3_ is also stimulated by visible light simultaneously, photogenerated electrons can jump from the valence band to the conduction band. After combining P_2_Mo_18_ and Fe_2_O_3_, electrons in the conduction band of Fe_2_O_3_ migrate to the conduction band of P_2_Mo_18_ with higher potential, while holes in the valence band of P_2_Mo_18_ migrate to the valence band of Fe_2_O_3_ with lower potential. However, the oxidation potential of the Fe_2_O_3_ valence band cavity is more damaging than that of OH^−^/·OH (1.99 V vs. NHE) and H_2_O/·OH (2.40 V vs. NHE). It cannot satisfy the overpotential conditions for generating · OH-reactive substances. This strongly contradicts the results of the capture trials. ·OH was discovered to be the active ingredient in the photocatalytic processes during the capture tests. As a result, the synthesized composite Fe_2_O_3_/P_2_Mo_18_ charge transfer pattern is more compatible with the unique Z-type heterojunction process. The photoexcited electrons of P_2_Mo_18_ immediately complex with the valence band holes of Fe_2_O_3_, maintaining the photogenerated holes on the HOMO of P_2_Mo_18_ and the photogenerated electrons in the conduction band of Fe_2_O_3_ engaged in photocatalytic activity. Based on this charge transfer mechanism, the oxidation potential of the valence band cavity in P_2_Mo_18_ can well match the requirement of ·OH radical generation. As the Fermi energy levels of P_2_Mo_18_ and Fe_2_O_3_ converge and eventually reach equilibrium, a built-in electrical field that can bend their energy band structures is formed between them, driving the photogenerated electrons of P_2_Mo_18_ to compound directly with the photogenerated holes of Fe_2_O_3_. During the degradation process of Cr (VI), the photogenerated electrons are the only active ingredient. The use of Z-type heterojunctions can not only effectively promote the separation and transfer of photogenerated charges but also maximize the retention of photogenerated electrons and holes with high chemical activity, resulting in a significant improvement in the photocatalytic performance of the catalyst. Thus, the primary reaction pathways for MB degradation and Cr (VI) reduction involving photogenerated electrons and holes are as follows.

(1) $${Fe}_{2}O_{3}/P_{2}{Mo}_{18}+hv\to {Fe}_{2}O_{3}(e^{-})/P_{2}{Mo}_{18}\left(h^{+}\right)$$

(2) $$P_{2}{Mo}_{18}\left(h^{+}\right)+OH^{-}/H_{2}O\to \cdot OH$$

(3) $$\cdot OH+MB\to CO_{2}+H_{2}O+intermediates$$

(4) $${Fe}_{2}O_{3}/P_{2}{Mo}_{18}+hv\to {Fe}_{2}O_{3}(e^{-})+P_{2}{Mo}_{18}(h^{+})$$

(5) $${Fe}_{2}O_{3}\left(e^{-}\right)+{Cr}_{2}O_{7}^{2-}+14H^{+}\to 2{Cr}^{3+}+7H_{2}O$$

## 3. Materials and Methods

### 3.1. Experimental Reagents

All of the chemical reagents were analytical grade and used without further purification. Deionized water was used throughout this study.

### 3.2. Preparation of P_2_Mo_18_

Na_2_MoO_4_·2H_2_O (0.207 mol) was dissolved in 225 mL of deionized water with 85% H_3_PO_4_, and then a particular amount of concentrated hydrochloric acid was added to acidify and heat the reflux for 10 h. After cooling the solution to room temperature, a certain amount of NH_4_Cl was added to crystallize and precipitate the P_2_Mo_18_ ammonium salt. The solid was dissolved again, and then NH_4_Cl was added for precipitation. This operation was performed numerous times and the sample was dried at 40 ℃ to provide pure P_2_Mo_18_.

### 3.3. Preparation of Fe_2_O_3_ Nanosheets

FeSO_4_·7H_2_O (0.278 g) and 0.246 g CH_3_COONa were dissolved in 40 mL of deionized water and stirred in the air for a few minutes. The solution was transferred to a reaction kettle preheated to 140 ℃. The reaction time was changed, and the heating times were 2 h, 6 h, 12 h, 18 h, and 24 h. The reaction kettle was cooled to ambient temperature before being opened, and the products were separated using a centrifuge. The samples were washed with deionized water and anhydrous ethanol before being dried in a 40 ℃ oven. The samples were transferred to crucibles and calcined for 2 h in a muffle furnace at 500 ℃ to produce Fe_2_O_3_ with various morphologies.

### 3.4. Preparation of Fe_2_O_3_/P_2_Mo_18_

P_2_Mo_18_ (1.0 g) was weighed and dissolved in 20 mL of water, then 0.01 g/0.03 g/0.05 g/0.08 g/0.10 g Fe_2_O_3_ was added and thoroughly mixed. After a while, dropwise additions of 20 mL of 0.1 mol/L aqueous tetrabutylammonium bromide solution were made, and the reaction was allowed to run for 24 h. After the reaction, the samples were centrifuged and washed with deionized water and ethanol to obtain composites with varying composite ratios, then placed into an oven at 50 ℃ and dried to obtain the final composites.

### 3.5. Material Characterizations

The structure and composition of the samples were characterized using FT-IR (Affinity-1 Shimadzu, Kyoto, Japan) and X-ray powder diffraction (XRD) collected on a Shimadzu powder diffractometer with Cu Ka radiation (λ = 0.15405 nm). X-ray photoelectron spectroscopy was used to determine the samples’ surface chemical state and elemental composition (XPS), (ESCALAB 250 Thermo Fisher Scientific, Waltham, MA, USA). The UV-vis diffused reflectance spectra were recorded using a (UV-2550 Shimadzu, Tokyo, Japan). The N_2_ adsorption–desorption isotherms were collected using a Micromeritics (ASAP 2460 analyzer Micromeritics, Norcross, GA, USA). The morphologies of the samples were observed using scanning electron microscopy (SEM), (SUPRA55 ZEISS, Oberkochen, Germany) transmission electron microscopy (TEM), (JEM-2100 JEOL, Tokyo, Japan), and the EIS test using the Princeton electrochemical workstation. A three-electrode system was used in the test. The mixed solution was 75 mL 0.05 mol/L potassium ferricyanide and 75 mL 0.1 mol/L potassium chloride. The cyclic voltammetry also used the above test conditions.

### 3.6. Photocatalytic Activity Evaluation

MB (100 mL, 20 mg/L) and Cr (100 mL, 50 mg/L) were transferred to the photocatalytic reactor as initial solutions. The amount of photocatalyst was 0.1 g. The photocatalyst was adsorbed using dark light for 30 min before photocatalysis, and then the xenon lamp was turned on for photocatalysis (illumination: 18.085 mW/cm^2^). Samples were collected every 10 min, for 9 times in total.

Three scavenger agents, isopropanol, triethanolamine, and p-benzoquinone, were used to test the active component holes, hydroxyl radicals, and superoxide radicals, respectively, with a dosage of about 0.05 mmol.

## 4. Conclusions

In summary, Fe_2_O_3_/P_2_Mo_18_ hybrid composites were successfully developed and produced utilizing the divisional synthesis approach. Differentiating the reaction duration may achieve good crystallinity, uniform size, and homogenous phase of Fe_2_O_3_ nanoparticles. Under simulated visible-light conditions, the Fe_2_O_3_/P_2_Mo_18_-5% composite material achieved a satisfactory degradation efficiency in the photocatalytic degradation of MB and reduction of Cr (VI), which was significantly better than that of single Fe_2_O_3_ or P_2_Mo_18_ samples, and the cyclic photocatalytic experiments demonstrated that the product possessed extremely high structural stability and performance stability. In particular, Fe_2_O_3_ complexed with P_2_Mo_18_ enhances the catalyst’s specific surface area and active sites, while the close contact at the interface allows photogenerated charges to migrate quickly. On the other hand, the results based on the energy band structure and active radical trapping led to the successful formation of Z-type heterojunctions in the presence of built-in electrical fields. The unique charge transfer mechanism can facilitate the separation and transfer of photogenerated charges, thus improving the quantum efficiency of the catalytic reactions. Therefore, compared with the previous literature (as shown in Tables S1 and S2), a substantial enhancement of the photocatalytic performance of the catalyst can be achieved.

## Data Availability

The data presented in this study are available on request from the corresponding author.

## Supplementary Materials

The following supporting information can be downloaded at: https://www.mdpi.com/article/10.3390/molecules28186671/s1, Figure S1. DRS spectra of Fe_2_O_3_; Figure S2: FI-IR spectra of the hybrid materials; Figure S3: N_2_ adsorption desorption isotherms and pore size distribution curves (Insert); Figure S4: FI-IR spectra of the hybrid materials with different reaction times of Fe_2_O_3_; Figure S5: UV-vis spectra of composites with different reaction times of Fe_2_O_3_; Figure S6: Nyquist (a) and Bode (b) plots of composites made from Fe_2_O_3_ with different reaction times; Figure S7: Photoluminescence spectra of composites with different reaction times of Fe_2_O_3_; Figure S8: The CV test diagram of polyoxoanion P_2_Mo_18_ (a), Fe_2_O_3_ (b); Table S1: The comparison of MB degradation activity of P_2_Mo_18_/Fe_2_O_3_-5% with literature; Table S2: The comparison of Cr reduction performance of P_2_Mo_18_/Fe_2_O_3_-5% with literature.

## Sample Availability

Samples of the compounds are not available from the authors.
