# Supplementary material for: Controllable Synthesis, Photocatalytic Property, and Mechanism of a Novel POM-Based Direct Z-Scheme Nano-Heterojunction α-Fe2O3/P2Mo18
Source: Molecules. 2023 Sep 18;28(18):6671. doi: 10.3390/molecules28186671 (PMC10536182; doi:10.3390/molecules28186671)
Supplement: Supplementary file 1 [file molecules-28-06671-s001.zip › molecules-2583274-supplementary.pdf]

Supporting materials

Controllable Synthesis Photocatalytic Property and Mechanism of a Novel Direct Z-scheme POMs-based Nano Heterojunction α-Fe_2O_3/P_2Mo_{18}

Yanlin Zhang,[a] MingYu Zhao,[a] Jubo Huang,[a] Nan Zhao,[a] Haihui Yu,*[a]

[a] School of Chemical Engineering, Northeast Electric Power University, Jilin 132012, P. R. China

*Correspondence: author. E-mail：hhyu@neepu.edu.cn

Characterization

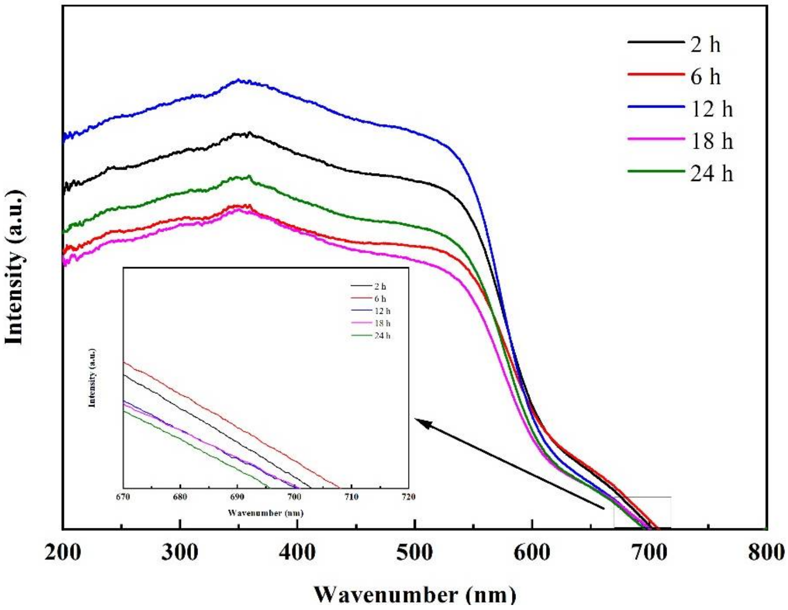

Fig. S1. DRS spectra of Fe_2O_3

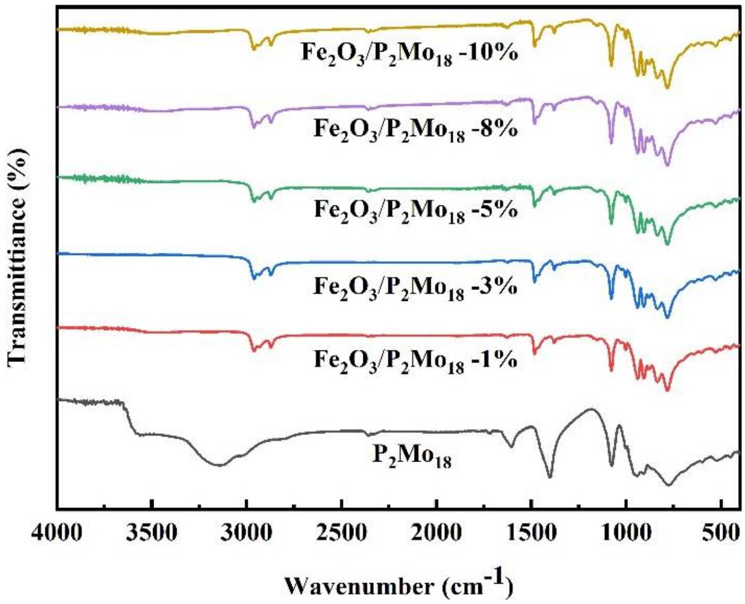

Fig S2. FI-IR spectra of the hybrid materials.

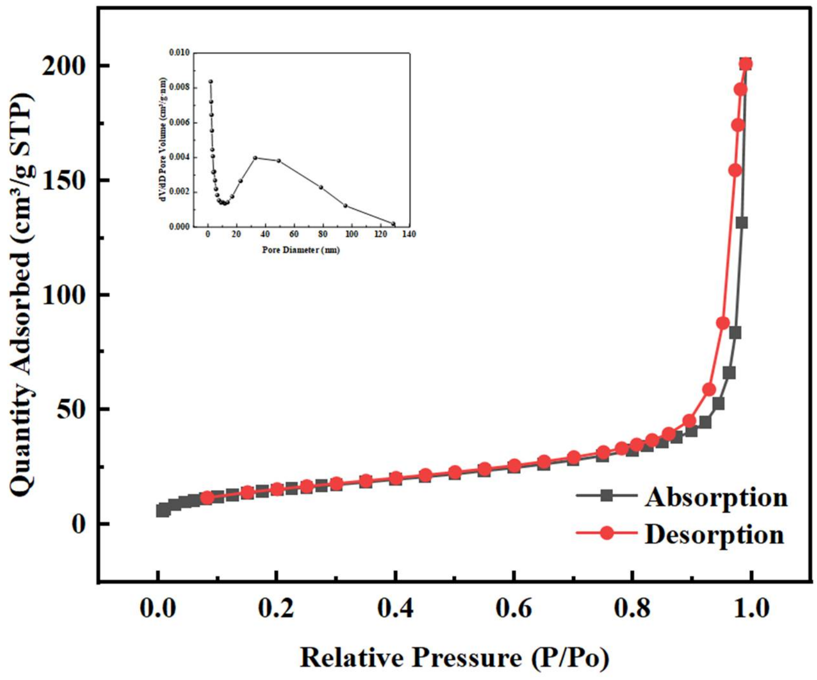

Fig. S3. N_2 adsorption desorption isotherms and pore size distribution curves (Insert)

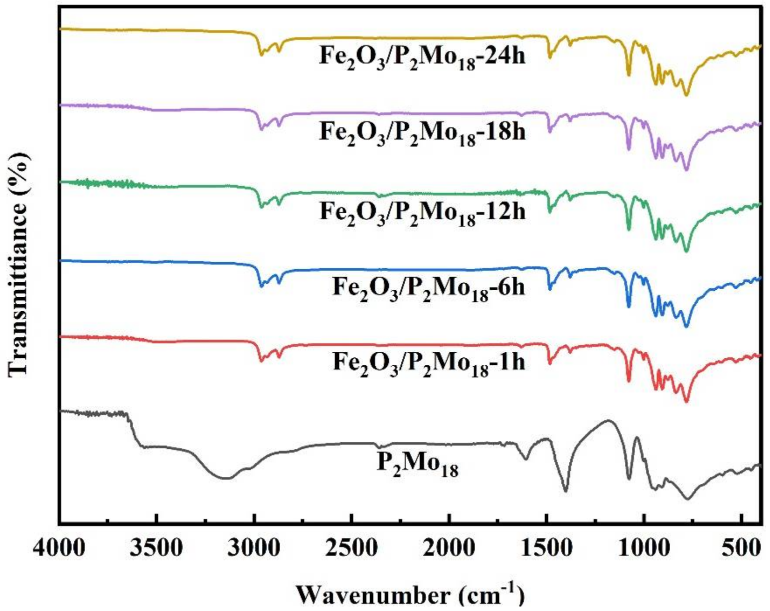

Fig. S4. FI-IR spectra of the hybrid materials with different reaction times of Fe_2O_3

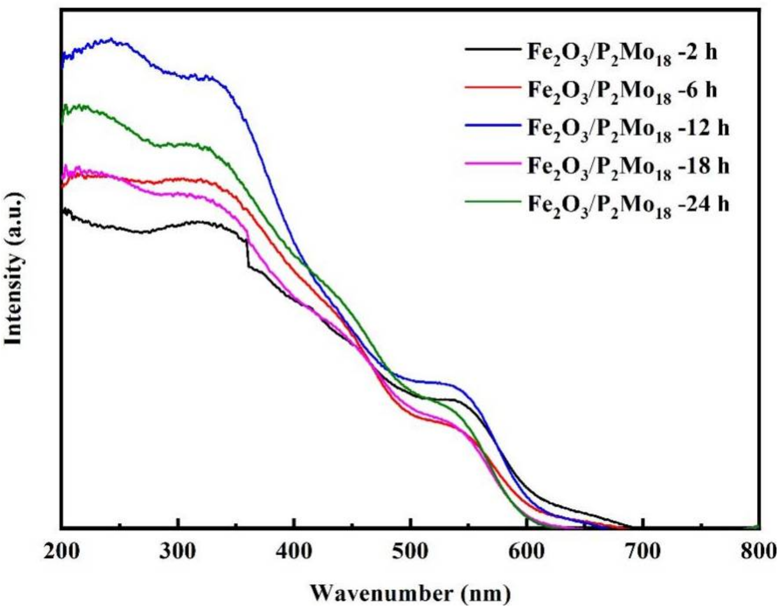

Fig. S5. UV-vis spectra of composites with different reaction times of Fe_2O_3

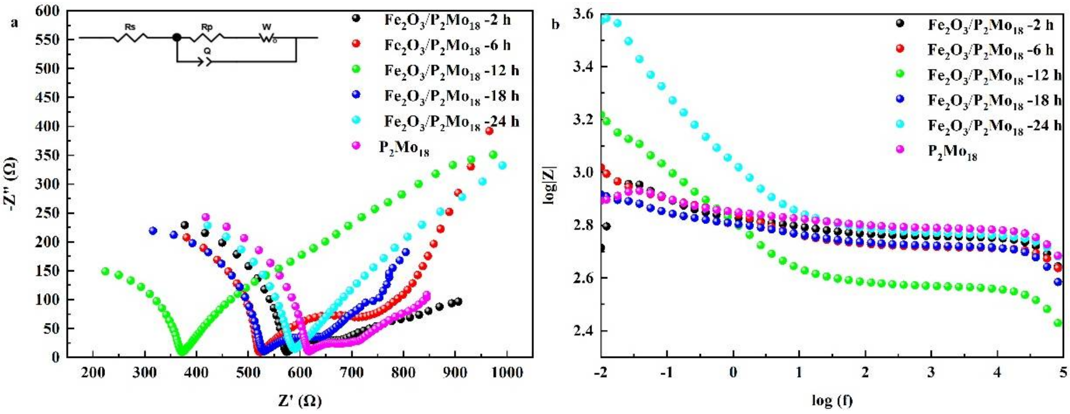

Fig. S6. Nyquist (a) and Bode (b) plots of composites made from Fe_2O_3 with different reaction times

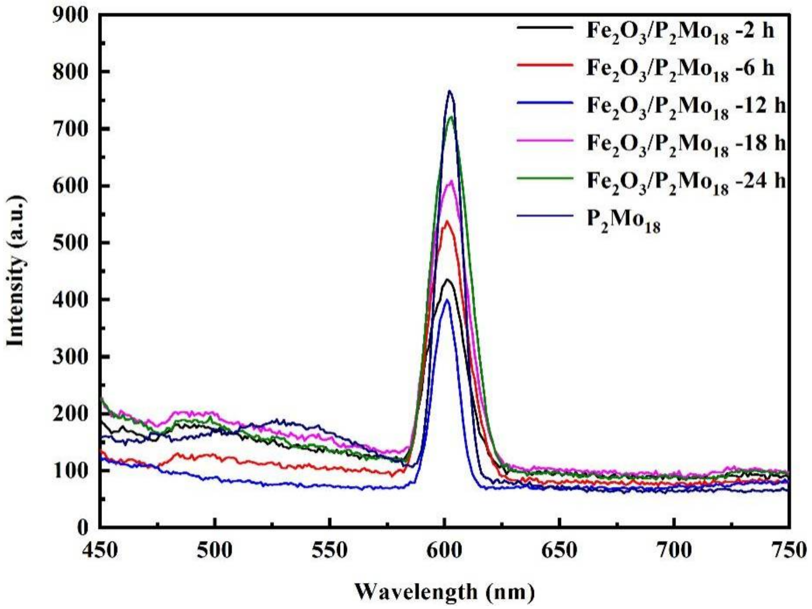

Fig. S7. Photoluminescence spectra of composites with different reaction times of Fe_2O_3

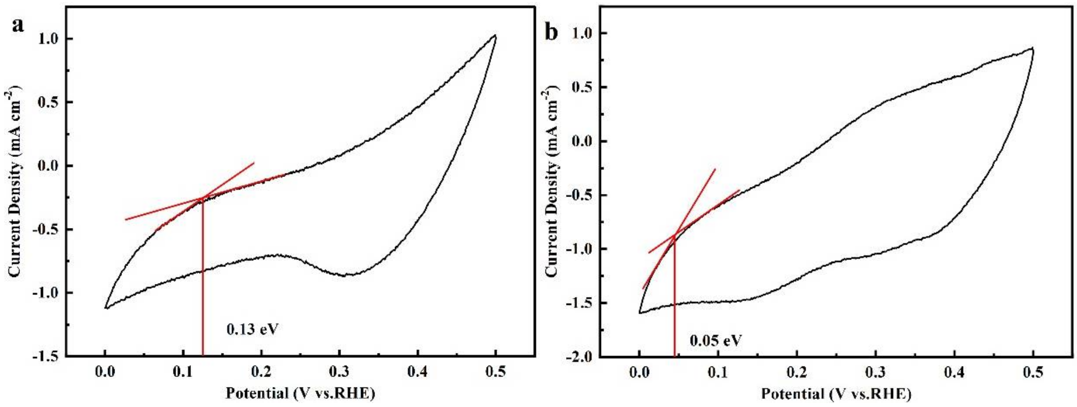

Fig. S8. the CV test diagram of polyoxoanion P_2Mo_{18} (a) ,Fe_2O_3 (b)

Table S1 The comparison of MB degradation activity of P_2Mo_{18}/Fe_2O_3-5% with literature.

Photocatalyst	Concentration ($mg\cdot L^{-1}$)	Dosage ($g\cdot L^{-1}$)	Time (min)	Remove (%)	Reference
Fe_2O_3	10	0.5	120	73	1
TiO_2	13.5	0.2	60	78	2
α-$Fe_2O_3/BaTiO_3$	20	0.5	180	94	3
α-Fe_2O_3/Bi_2WO_6	5	0.2	25	98	4
K_8/EuW_{10}	2	0.25	2880	91	5
CdS@ZIF-8	10	1	120	72	6
ZnO/CeO_2	10	0.5	60	70	7
MoS_2/ZnO	10	0.05	180	93	8
Fe_2O_3/P_2Mo_{18}	20	0.1	90	98.9	This work

Table S2 The comparison of Cr reduction performance of P_2Mo_{18}/Fe_2O_3-5% with literature.

Photocatalyst	Concentration ($mg \cdot L^{-1}$)	Dosage ($g \cdot L^{-1}$)	Time (min)	Remove (%)	Reference
$BiVO_4/FeVO_4$ @rGO	20	0.025	90	90.9	9
Co_3O_4/g-C_3N_4	15	0.02	150	92.6	10
TiO_2	10	0.01	180	62	11
TiO_2/SiO_2	3	0.1	180	93	12
Fe_3O_4–ZnAl-LDH and TiO_2	20	0.1	420	98	13
α-Fe_2O_3/g-C_3N_4	10	0.1	150	98	14
AgI/$BiVO_4$	15	0.02	100	71	15
$Ag_2CO_3/BiVO_4$	15	0.02	150	70	16
Fe_2O_3/P_2Mo_{18}	50	0.1	90	95.9	This work

Reference:

[1] Zong M, Song D, Zhang X, et al. Environmental Science and Technology, 2021, 55(1):677-688

[2] Maleki H, Bertola V. ACS Applied Nano Materials, 2019, 2(11):7237-7244

[3] Yahia B, Faouzi S, Mohamed T. Journal of Photochemistry and Photobiology A: Chemistry, 2023, 439: 114634.

[4] Wang W, Zhao W, Zhang H, et al. Chinese journal of catalysis, 2021, 42(1): 97-106.

[5] Tong L, Wang Z, Xia C, et al. Journal of Physical Chemistry B, 2017, 121(46):10566-10573

[6] Ankur M, Mala N, Shanid M, et al. Acs Omega, 2018, 3(7):8288-8308

[7] Rajendran S, Khan M M, Gracia F, et al. Scientific reports, 2016, 6(1): 31641.

[8] Chang Y C, Lin Y W, Lu M Y. Materials Chemistry and Physics, 2021, 266: 124560.

[9] Yang R, Zhu Z, Hu C, et al. Chemical engineering journal, 2020, 390: 124522.

[10] Zhao W, Li J, She T, et al. Journal of Hazardous Materials, 2021, 402: 123839.

[11] Preethi R, Singh S. Journal of Environmental Management, 2023, 326: 116831.

[12] Eddy D R, Ishmah S N, Permana M D, et al. Catalysts, 2020, 10(11): 1248.

[13] Yang Y, Li J, Yan T, et al. Journal of colloid and interface science, 2020, 562: 493-501.

[14] Xiao D, Dai K, Qu Y, et al. Applied Surface Science, 2015, 358: 181-187.

[15] Huang Z, Dai X, Huang Z, et al. Chemosphere, 2019, 221: 824–833.

[16] Yuan L, Weng B, Colmenares J C, et al. Small, 2017, 13(48): 1702253.
